# Quantitative assessment of the spatial heterogeneity of tumor-infiltrating lymphocytes in breast cancer

**DOI:** 10.1186/s13058-016-0737-x

**Published:** 2016-07-29

**Authors:** Nikita L. Mani, Kurt A. Schalper, Christos Hatzis, Ozlen Saglam, Fattaneh Tavassoli, Meghan Butler, Anees B. Chagpar, Lajos Pusztai, David L. Rimm

**Affiliations:** 1 Department of Pathology, Yale School of Medicine, Yale University, 310 Cedar St., PO Box 208023, New Haven, CT 06520-8023 USA; 2 Section of Medical Oncology, Department of Internal Medicine, Yale School of Medicine, Yale University, New Haven, CT USA; 3 Department of Pathology, Moffitt Cancer Center, Tampa, FL USA; 4 Department of Surgery, Yale School of Medicine, Yale University, New Haven, CT USA

**Keywords:** Immunofluorescence, Tumor microenvironment, Breast cancer, T cells, B cells, Stroma

## Abstract

**Background:**

Tumor-infiltrating lymphocyte (TIL) count in breast cancer carries prognostic information and represents a potential predictive marker for emerging immunotherapies. However, the distribution of the lymphocyte subpopulations is not well defined. The goals of this study were to examine intratumor heterogeneity in TIL subpopulation counts in different fields of view (FOV) within each section, in different sections from the same biopsy, and between biopsies from different regions of the same cancer using quantitative immunofluorescence (QIF).

**Methods:**

We used multiplexed QIF to quantify cytokeratin-positive epithelial cells, and CD3-positive, CD8-positive and CD20-positive lymphocytes in tissue sections from multiple biopsies obtained from different areas of 31 surgically resected primary breast carcinomas (93 samples total). Log2-transformed QIF scores or concordance and variance component analyses with linear mixed-effects models were used. Cohen’s kappa index [k] of high versus low scores, defined as above and below the median, was used to measure sample similarity between areas.

**Results:**

We found a strong positive correlation between CD3 and CD8 levels across all patients (Pearson correlation coefficient [CC] = 0.827). CD3 and CD8 showed a weaker but significant association with CD20 (CC = 0.446 and 0.363, respectively). For each marker, the variation between different FOVs in the same section was higher than the variation between sections or between biopsies of the same cancer. The intraclass correlation coefficients (ICC) were 0.411 for CD3, 0.324 for CD8, and 0.252 for CD20. In component analysis, 66–69 % of the variance was attributable to differences between FOVs in the same section and 30–33 % was due to differences between biopsies from different areas of the same cancer. Section to section differences were negligible. Concordance for low versus high marker status assignment in single biopsies compared to all three biopsies combined yielded k = 0.705 for CD3, k = 0.655 for CD8, and k = 0.603 for CD20.

**Conclusions:**

T and B lymphocytes show more heterogeneity across the dimensions of a single section than between different sections or regions of a given breast tumor. This observation suggests that the average lymphocyte score from a single biopsy of a tumor is reasonably representative of the whole cancer.

**Electronic supplementary material:**

The online version of this article (doi:10.1186/s13058-016-0737-x) contains supplementary material, which is available to authorized users.

## Background

With the advent of effective immunotherapies for cancer, there has been renewed interest in the tumor immune infiltrate. A number of studies have shown the prognostic value of the presence of tumor-infiltrating lymphocytes (TILs) in a range of cancer types [1–3]. Increased levels of CD8+ cytotoxic T cells, in particular, has been associated with both better outcome [4] and response to programmed cell death receptor 1 (PD-1) therapy in melanoma [5] and microsatellite instable-high colorectal carcinoma [6]. Similarly, patients who express high levels of programmed cell death ligand 1 (PD-L1) display prominent TILs and have been shown to respond more favorably to immunostimulatory therapy [7].

The spatial organization of the infiltrating lymphocytes is not well defined and represents a potential confounding factor in the assessment of TILs. The constituents of anti-tumor immunity such as macrophages, natural killer (NK) cells, mast cells, and lymphocytes are organized in different spatial patterns both in and around tumors, presumably representing the a range of immune responses [8]. Tertiary lymphoid structures, analogous to structures of the lymph node with germinal centers, dendritic cells, highly proliferative B cells, and high endothelial venules, have been noted in a variety or neoplastic malignancies [9] and tend to correlate with more favorable outcomes [10]. In contrast, T cells may infiltrate in either a sporadic or in a more uniform manner [11]. These variable patterns of immune cell infiltration represent a highly heterogeneous appearance and present a challenge to reproducibly quantifying and meaningfully defining TILs.

Traditionally, TILs, like most other cancer biomarkers, have been scored by pathologists using standard hematoxylin and eosin (H&E)-stained tissue sections. Though conventional and efficient, this method is limited in its semiquantitative feature of measurement and is prone to high interscorer subjectivity and variability. The issue of assessment of TILs has been addressed by an international consortium of pathologists in a round robin study [12]. The initial efforts showed only modest reproducibility, but subsequent studies where scoring aids were used resulted in good concordance between pathologists [13]. While this method appears be sufficient for pathologist-based assessment, it is nonquantitative, cannot discriminate between TIL subsets, and would be insufficient for assessment of the distribution of TILs within a tumor. In addition, the possible impact of intratumor heterogeneity for use of TILs as tissue biomarker in breast cancer remains unknown. Here, we have used a previously validated multiplexed quantitative immunofluorescence (QIF) approach [14] to measure TILs in prospectively collected biopsies from three different regions of resected breast tumors. We describe the distribution of different TIL phenotypic markers, both within separate regions of a tumor and within a given tissue section, and then apply statistical analysis to determine the degree of variance for each marker across the tumor.

## Methods

### Tissue collection and patient cohort

Newly diagnosed breast cancer patients with primary invasive tumors greater than 1.5 cm who had not undergone neoadjuvant therapy and who were undergoing surgery for their breast cancers at Yale-New Haven Hospital were eligible for this study (see Table 1). Written informed consent was obtained from 33 patients who enrolled in the study. All tissue was used in accordance with Yale Human Investigational Committee protocol number 1207010483 and reviewed and approved by this committee prior to collection.

**Table 1:** Tissue sampling and heterogeneity assessment

Characteristic	Number	%
Age at diagnosis		
≥50	23	69.7
<50	10	30.3
Histological grade		
1	0	0
2	20	60.6
3	13	39.4
Tumor size		
<2 cm	6	18.2
2–5 cm	25	75.8
≥5 cm	2	6.1
ER status		
ER positive	23	69.7
ER negative	10	30.3
PgR status		
PgR positive	19	57.6
PgR negative	14	42.4
HER2 status		
HER2 positive	5	15.2
HER2 negative	28	84.8

Whole tissue serial sections of core biopsies from different regions of the same cancer were prepared and multiple fields of view (FOV) were assessed in each section

*ER* estrogen receptor, *PgR* progesterone receptor. *HER2* human epidermal growth factor receptor 2

**Fig. 1:**
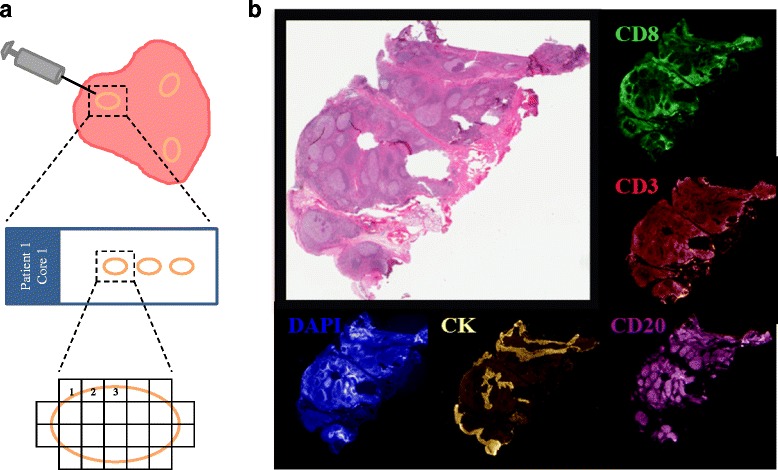
Average AQUA® scores for tonsil whole tissue control samples. **a** Whole tissue serial sections of core biopsies from different regions of the same cancer were prepared and multiple fields of view (FOV) were assessed in each section. **b** Hematoxylin staining of tumor-infiltrating lymphocytes (TILs) compared to CD3, CD8, CD20, cytokeratin, and DAPI staining under fluorescence microscopy from a multiplexed tonsil control slide

At the time of surgery, tissue was first sampled by the pathologist for diagnostic purposes. Residual nondiagnostic tumor was used in this study. There was insufficient residual tissue for research purposes in two patients. In 26 patients, a 2-mm punch biopsy specimen was taken from each of three separate tumor regions, at roughly the 2, 6, and 10 o’clock positions; in the remaining five patients, biopsies could only be obtained from two separate areas. Each biopsy was formalin fixed and paraffin embedded into separate blocks according to standard pathology procedure. Each block was evaluated by H&E staining for the presence of both TILs and tumor tissue (carcinoma) by a board-certified pathologist (OS). Slides for quantitative immunofluorescence (QIF) studies contained two to six serial sections of the same biopsy. A total 15–114 20× magnification fields of view (FOV) were scored under the fluorescence microscope, corresponding to 6–50 FOVs per section (mean 19.5). Figure 1a shows a schema of the assessment strategy.

### Multiplexed quantitative immunofluorescence staining for TILs

Protein detection of CD3, CD8, CD20, cytokeratin, and 4’,6-diamidino-2-phenylindole (DAPI) were simultaneously quantified on the same slide for every patient, as previously detailed by Brown et al., [14]. Briefly, fresh cuts of whole tissue sections were deparaffinized and rehydrated before undergoing antigen retrieval using an EDTA buffer (pH = 8) for 20 minutes at 97 °C (PT module, Lab Vision, Thermo Fisher Scientific, Waltham, MA, USA). Slides were then incubated with dual endogenous peroxidase block (Dako, Glostrup, Denmark) for 10 minutes to block endogenous peroxidase activity and then incubated with 0.3 % bovine serum albumin in a 0.05 % Tween solution for 30 minutes to block nonspecific antigens. Fluorescent staining for pancytokeratin, CD3, CD8, and CD20 was performed using a sequential multiplexed protocol with different isotype-specific primary antibodies. Antibodies against these targets were used to detect epithelial tumor cells (cytokeratin, clone M3515, Dako), all T lymphocytes (CD3 IgG, clone E272, Novus Biologicals, Littleton, CO, USA), cytotoxic T lymphocytes (CD8 IgG1, clone C8/144B, Dako), and B lymphocytes (CD20, IgG2a, clone L26, Dako). All nuclei were then tagged with DAPI (Life Technologies, Carlsbad, CA, USA). Secondary antibodies conjugated to horseradish peroxidases (HRPs) and specific to each primary antibody isotype were used (anti-rabbit EnVision, Dako; anti-mouse IgG1, eBioscience, San Diego, CA, USA; anti-mouse IgG2a, Dako), while tyramide-bound fluorophores were added to bind to the HRPs (biotinylated tyramide, PerkinElmer, Waltham, MA, USA; streptavidin-Alexa750, Life Technologies; TSA™Plus fluorescein-tyramide, PerkinElmer; cyanine 5, PerkinElmer, respectively). A fluorophore-conjugated goat anti-rabbit secondary antibody was used against the cytokeratin antibody (Goat anti-Rabbit Alexa546, Life Technologies). Residual, unbound HRPs were blocked between incubations with a 0.15 % hydrogen peroxide benzoic hydrazide solution.

Slides were stained in three batches with a LabVision autostainer, in which samples from 10 to 11 patients were stained in each run. All biopsies from the same tumor were stained in the same batch to reduce experimental variability of expression of each target within patient samples. Morphologically normal human tonsil whole tissue sections were included in each batch as lymphocytic-positive control slides and to account for any variability in protein expression between batches. Additional file 1: Figure S1 shows small batch to batch variation for each marker.

### Fluorescence measurement and scoring

Quantitative measurement of fluorescent signal was obtained using automated quantitative analysis (AQUA®) technology (Genoptix, Inc., Carlsbad, CA, USA), which allows for objective and accurate measurement of protein expression within predetermined tumor and/or other subcellular compartments, as previously described [14]. FOVs, or areas of interest, on each slide were selected for in a preliminary low-resolution scan based on nuclear DAPI staining. Each FOV was then captured at high-resolution fivefold, with fluorescent wavelengths matching the five fluorophores used during staining (DAPI, FITC, Cy3, Cy5, and Cy7).

In order to accurately quantify the signal intensity of the emission wavelengths in each fluorescent channel with AQUA® software, areas not expressing invasive breast carcinomas as demonstrated by cytokeratin staining [e.g., normal breast tissue, ductal carcinoma in situ (DCIS)] were excluded from analysis, as well as any preparative artifacts (e.g., folded or damaged tissue). QIF scores for each FOV were generated for each channel by dividing the target marker pixel intensity by the total tissue area in that particular FOV (as defined by DAPI staining). Scores were normalized to exposure time and bit depth during time of capture to allow proper comparison across all samples. We used the average QIF scores of a given marker from all FOV in a given section to represent marker expression at the section level. We calculated the average QIF score from all sections from a single biopsy to represent marker expression at the biopsy level.

### Statistical analysis

For statistical analysis, QIF scores for each marker were log2 transformed to minimize the possible effects of the differential score distribution across cases. Pearson’s correlation coefficients (CC) and intraclass correlation coefficients (ICC) were calculated for each marker to measure the similarity of marker scores between FOVs, section to section in the same biopsy, and between biopsies from the same tumor. The variance components analysis used a linear mixed effect (LME) regression model was used to estimate the contribution of each source of variation to the total variation observed. Biopsies were also categorized into lymphocyte low versus high groups using the median for each lymphocyte subtype marker. Cohen’s kappa coefficient (k) was then used to calculate the concordance in TIL category obtained from assessing a single, randomly chosen biopsy versus the averaged results from all three biopsies from a given cancer. Statistical analyses were performed using the R v3.2.2 statistical platform (R Foundation for Statistical Computing) and GraphPad Prism v6.0 for Windows (GraphPad Software, Inc, San Diego, CA, USA).

## Results

### Validation of quantitative immunofluorescence staining

Systematic staining of serially sectioned, morphologically normal human tonsil tissue was used as positive control. It revealed the expected levels and expression patterns of CD3, CD8, and CD20 (Fig. 1b). CD3 and CD8 showed a membranous staining pattern and were located predominantly in areas outside of the follicular germinal centers. Staining with CD20 showed a membranous cellular staining of cell exclusively within the germinal centers that are typically rich in B cells. Cytokeratin positivity was found in the squamous epithelium lining of the crypts, characteristic of tonsil tissue. QIF scores for CD3, CD8, and CD20 showed high concordance and remained reproducible for each marker between tonsil slides stained in different batches indicating limited interbatch variation (Additional file 1: Figure S1).

### Quantitative assessment of TILs by immunofluorescence of breast cancer

**Fig. 2:**
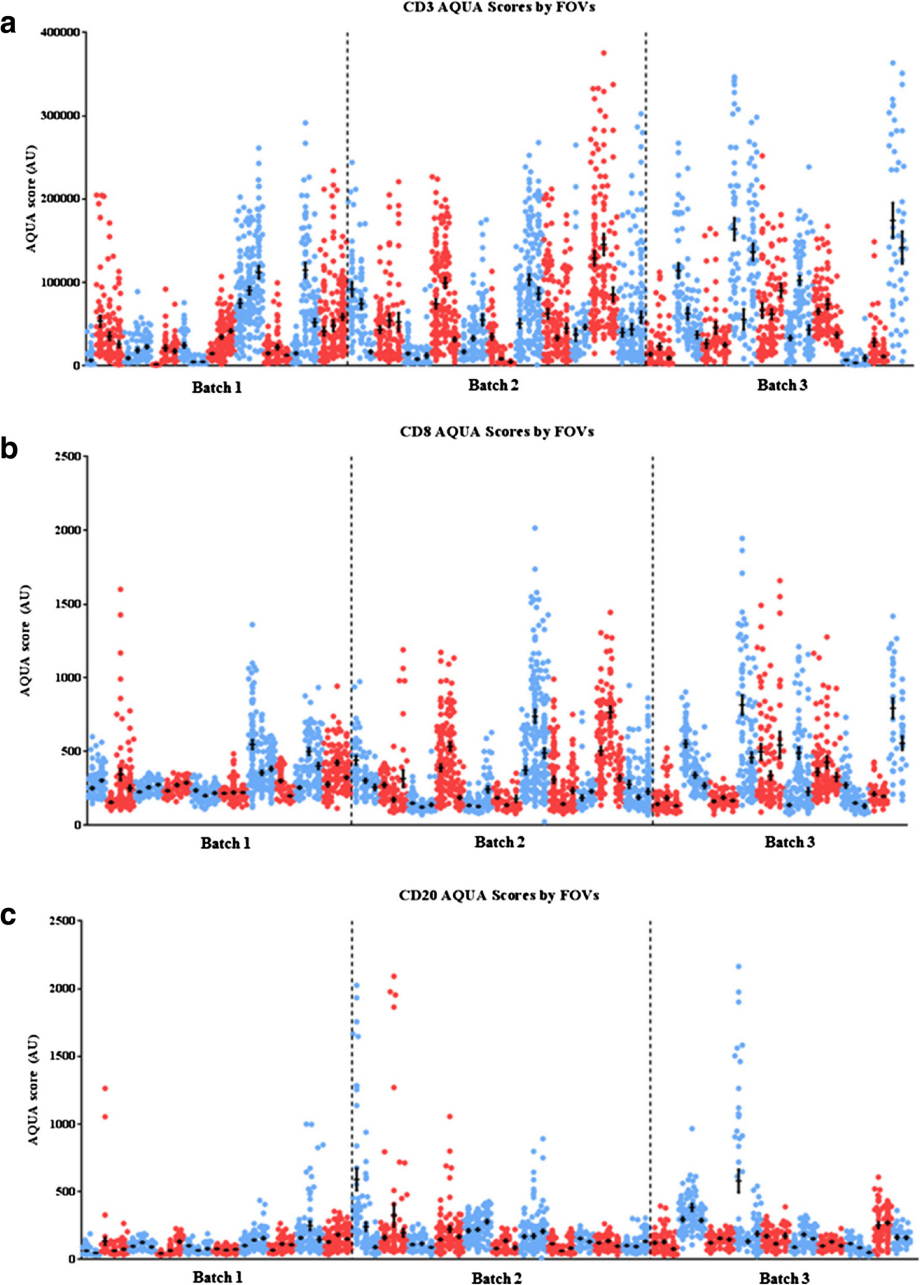
Distribution of AQUA® QIF scores of CD3 (T cells, **a**), CD8 (cytotoxic T cells, **b**), and CD20 (B lymphocytes, **c**) markers across three cores from 31 individual breast tumors. The 33 patient cases were randomly distributed into three staining batches completed on consecutive days where all three biopsies per tumor were stained within the same batch. The three core biopsy sets from each tumor are grouped together sequentially and are represented by the same color. Each tumor (three biopsy set) is color-coded with alternating *red* and *blue dots* for visual clarity between patients. Each dot represents a QIF score from a single field of view (FOV) from each biopsy. QIF scores are expressed as arbitrary units of fluorescence (AU) using the AQUA® algorithm. The mean score and standard error of the mean (SEM) for each core are indicated with a *black dot/bar*, respectively

**Fig. 3:**
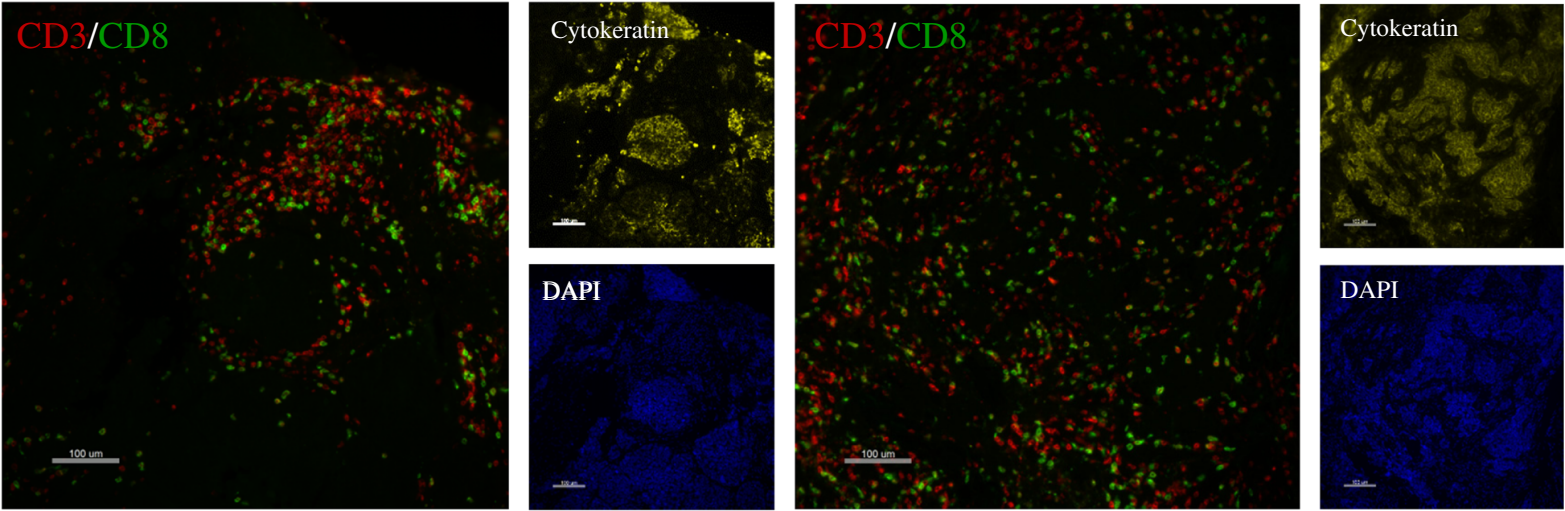
Representative immunofluorescence images of CD3 and CD8 in one patient. FOVs were compared between different core biopsies of the same patient. Spatial distribution of CD3+ and CD8+ T cells shows both random distribution of T cells among the various margins between and around epithelial cells and also aggregations of T cells into clusters near tumors

QIF scores for each marker were generated on a per FOV basis on a total of 5019 FOVs from 31 cases as described above. The distribution of all FOV scores for each biopsy is shown in Fig. 2, where biopsies were grouped by patient and by batch. CD3-positive lymphocytes followed by CD8 cells showed the highest levels and the greatest dynamic range within a biopsy and between tumors. Visually, both CD3 and CD8 cells were arranged in a random distribution within and around epithelium-expressing tumor cells while occasionally aggregating into clusters around tumors, as depicted by Fig. 3. In contrast, CD20 cells had the lowest frequency among the lymphocyte subpopulations that we examined and showed a unique pattern of focal positivity with strong positivity in certain areas and very low scores in most other areas within the same biopsy (Additional file 1: Figure S2). These focal, high CD20 B cell clusters are most likely tertiary lymphoid structures in the tumor microenvironment. While average CD3 and CD8 scores were largely concordant across different biopsies from the same areas or same cancer, CD20 scores due to the focal positivity were less concordant between biopsies (Fig. 2 and see below). Representative immunofluorescence images showing the heterogeneity in CD3, CD8, and CD20 expression levels can be observed in Additional file 1: Figure S3, where each marker is represented by a different patient.

**Fig. 4:**
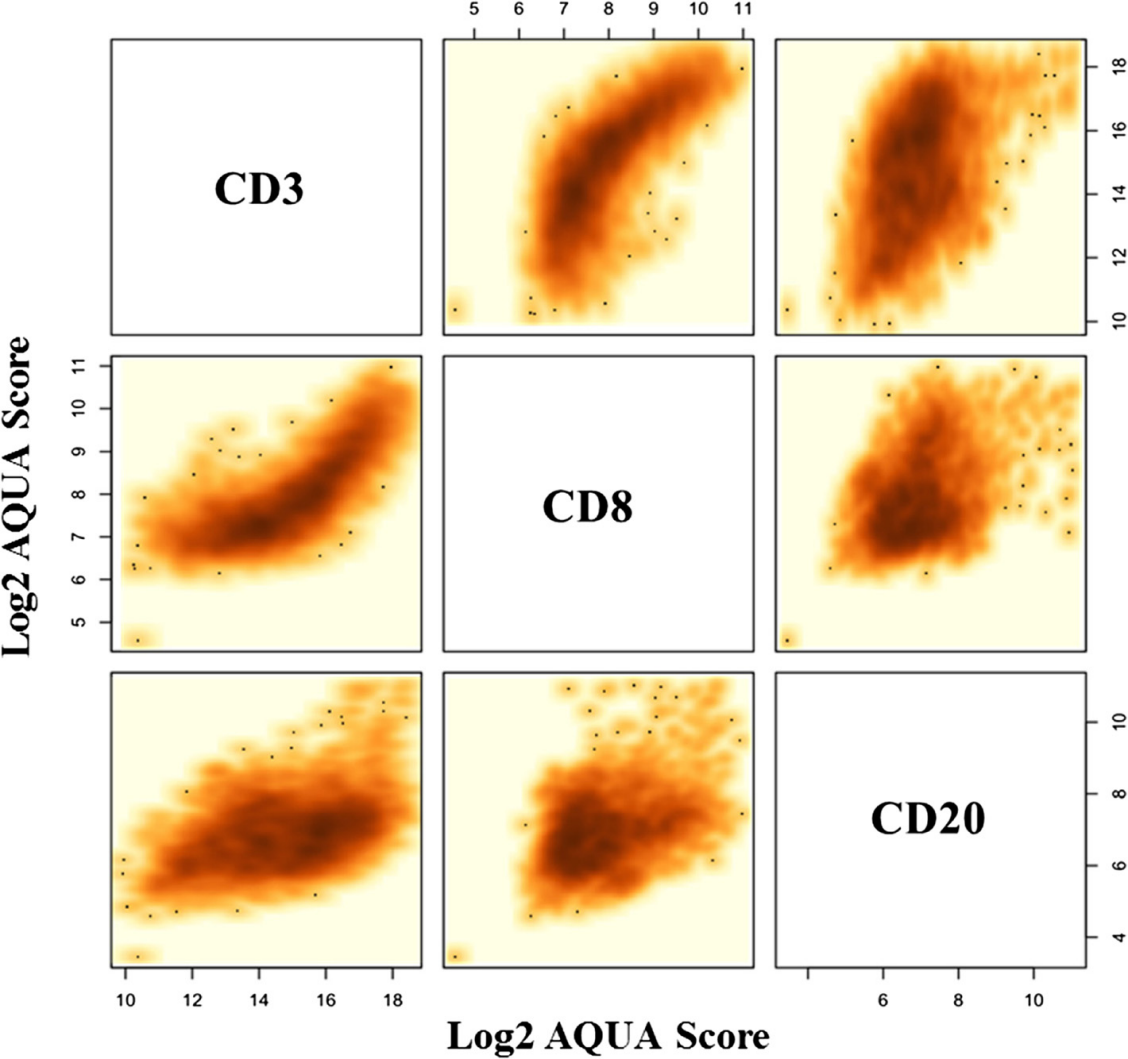
Correlation between TIL markers in breast cancer. AQUA® scores for the three markers (CD3, CD8, CD20) were log2 transformed and compared to each other on a FOV basis. Positive correlation exists between all three markers. The strongest correlation was between CD3 and CD8 (Pearson correlation coefficient [CC] = 0.827). The correlation between CD3 and CD20 was 0.446 and between CD8 and CD20 it was 0.363

As expected, there was a strong positive correlation between CD3 and CD8 expression levels in all biopsies with a Pearson correlation coefficient of 0.827. The expression of CD3 and CD8 showed a weaker association with CD20, with estimated correlation coefficients of 0.446 and 0.363, respectively (Fig. 4).

### Intratumor variability of TIL subpopulations

**Fig. 5:**
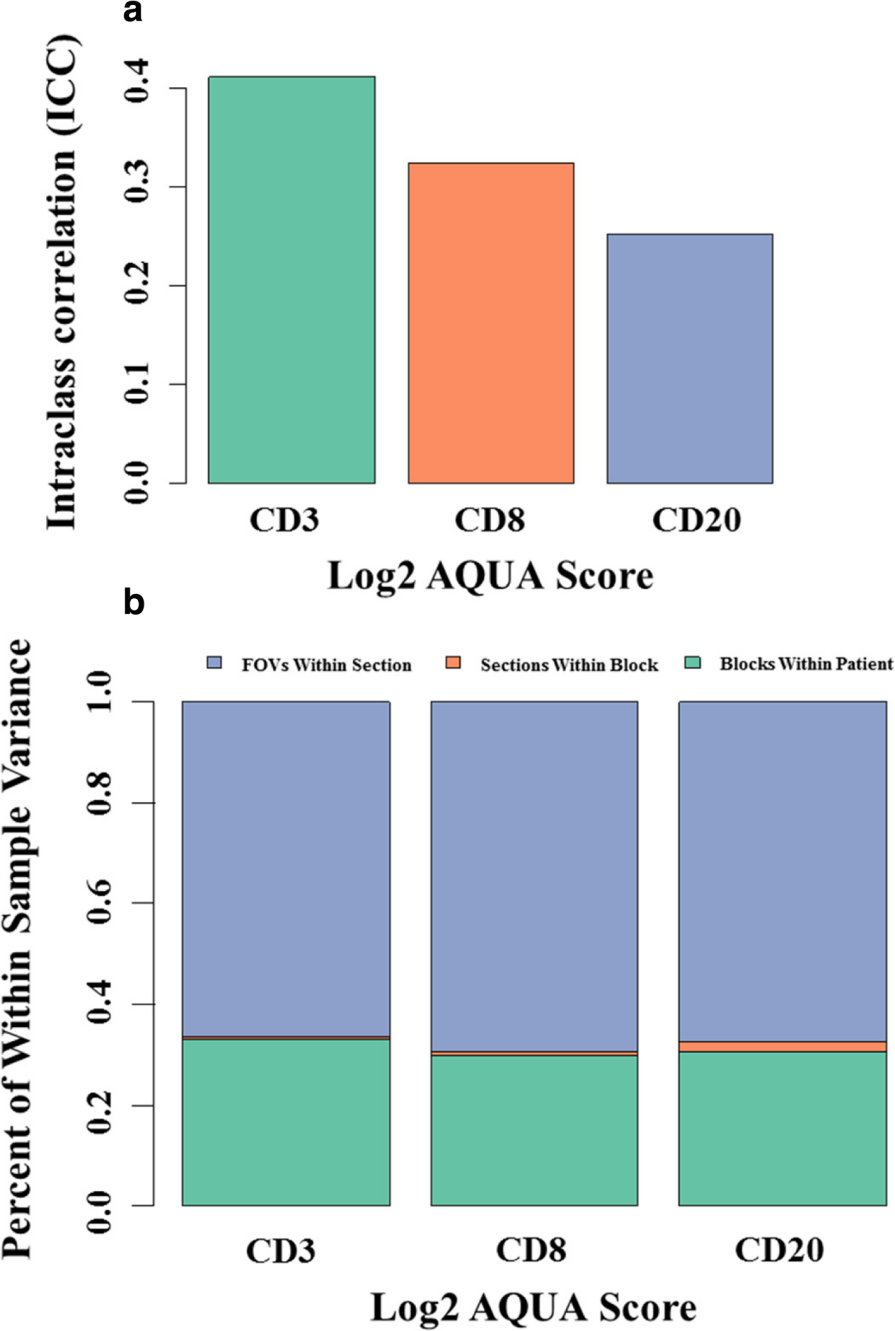
Variance of TILs scores in breast cancer. **a** The variance for each marker within cores of the same tumor are expressed as intraclass correlation coefficients (ICC). ICC was of 0.411 for CD3, 0.324 for CD8, and 0.252 for CD20. **b** The analysis included the marker change between FOVs in the same tumor section (*blue*), between serial sections of the same core (*orange*) and between cores of the same tumor (*green*). Variance components of TILs scores from the same cancer indicate that 66–69 % of the variance is attributable to signal differences between fields of view of the same section, 30–33 % is due to differences between biopsies from different areas of the cancer and <2 % is due to differences between serial cuts from the same biopsy

To determine the extent of heterogeneity in TILs distribution in breast cancer, each lymphocyte subpopulation was assessed for the degree of variance between FOVs, between serial sections, and between biopsies of the same tumor (Fig. 5). Intraclass correlation coefficient (ICC) assessment of the log2-transformed QIF scores revealed an ICC of 0.441 for CD3, 0.324 for CD8, and 0.252 for CD20 (Fig. 5a). These data suggest that 44.1 % of the variation observed in CD3 signal intensity can be attributed to the differences between biopsies from different regions of a given patient’s tumor, but that the remaining 59.1 % is due to the heterogeneity of the marker between FOVs of the same tumor core. Likewise, 32.4 % of variation in CD8 is due to intercore differences while the remaining 67.6 % is due to intraslide variation. Most strikingly, only 25.2 % of the variation detected in CD20 is accounted for by the differences seen between cores and the majority 74.8 % is due to the differences seen within each slide, confirming the prominent field heterogeneity of B cells in breast cancer.

When sources of variation in a single cancer were examined, variance components analysis using a linear mixed-effects regression showed that for all three markers, 30–33 % of the variation in expression levels is a result of between-biopsy differences while 66–69 % of variation is due to variable scores between FOVs in the same section. Only 2 % or less of the variation is due to differences in scores between serial sections of the same biopsy (Fig. 5b).

We also calculated average QIF scores for each biopsy and used the median score of the entire population as threshold to assign high TIL (i.e., above median) and low TIL (i.e., below median) categories to each biopsy. To test whether a single biopsy can provide a representative score for the entire cancer, we calculated Cohen’s kappa coefficient (k) for agreement in TIL classifications (high versus low) obtained from one random biopsy and from the average score from all three biopsies of the same cancer (only the 26 patients with all three viable biopsies were included in this analysis). Kappa values were 0.705 for CD3, 0.655 for CD8, and 0.603 for CD20 indicating very good to excellent agreement.

To assess the representivity of any given core, we assumed the mean of three cores to be representative of the tumor, then determined how often any given core was more than one standard deviation from the mean. Since the analysis is continuous, it is hard to prove that this method defines the number of cores that could be nonrepresentative of the whole case, but is an estimate of that parameter. We found only two out of the 89 total cores showed CD3 FOV mean score outside of the standard deviation of the overall average CD3 score for the case. By this definition, any given core is 97.8 % likely to be representative for CD3 expression. For CD8, none of the 89 cores in question had CD8 FOV mean scores outside of each the standard deviation for each patient and only one core had a mean CD20 score outside of the patient’s combined CD20 scores (98.9 % accurate).

## Discussion

There is growing interest in quantifying and reporting total TIL count and TIL subpopulations in clinical specimens due to their prognostic and possibly predictive value for immunotherapies [8]. An international group of pathologists recently proposed a standardized method to assess TILs with the intent to facilitate including this parameter in routine pathology reports [12]. The preferred way to establish the histologic diagnosis of breast cancer is core needle biopsy. These biopsies yield small amounts of tissue and sampling bias may influence biomarker results obtained from needle biopsies. Many clinically relevant markers [estrogen receptor (ER), human epidermal growth factor receptor 2 (HER2), Ki67] can be determined reliably from small biopsies of a cancer. However, markers that display high intratumor heterogeneity may yield results that are not representative of the entire tumor. The purpose of this study was to examine intratumor heterogeneity of TIL subpopulations. We examined the distribution of TIL populations using quantitative immunofluorescence and measured intratumor heterogeneity at three levels: heterogeneity between microscopic (20× magnification) FOVs in the same section, heterogeneity between average TIL scores between sections of the same biopsy, and average scores between biopsies from the same cancer.

This method of analysis led us to a few key observations. The first is that TILs, predominantly as measured by CD3 or CD8, show reasonable homogeneity between cores from specimens that were biopsied in three spatially distinct locations. The mean levels of either CD3 or CD8 (Fig. 2) show good concordance across the three cores from the same specimen. Thus, while heterogeneity was broad within a specimen, the overall assessment of a given core, in most cases, appears to be representative of the mean for the entire case. This is reassuring in that it suggests that a single core biopsy, as often obtained in a clinical setting, may be sufficient to represent the TILs from the entire tumor. We also observed that B cells exist in small clusters in the tumor microenvironment. The concept of small clusters of B cells, tertiary lymphoid structures, is well established [15]. This anatomical feature of B cell infiltrates explains the greater intratumor heterogeneity of CD20 scores.

Our study is not without limitations. For instance, the use of fluorescence intensity scores that incorporate the intensity of each marker per cell prevents the accurate determination of the absolute number of lymphocytes and reduces our capacity to compare the relative abundance of each cell subpopulation. In addition, the inclusion in our study of cases with different biological breast cancer subtypes [(ER+, HER2+, triple-negative breast cancer (TNBC)], without uniform treatment and relatively short follow-up limits our capacity to explore the clinical implications of TILs heterogeneity in breast cancer. Finally, we have used AQUA® technology, which averages intensity over an FOV rather than actually counting each cell. While these values are not equal and have the potential to assess very different variables, we have shown a comparison of AQUA® scores and CD8 and CD20 cell counts in a sample lung TMA in Additional file 1: Figure S4.

## Conclusions

In summary, we have applied an objective and reproducible immunofluorescence-based assay to quantify the distribution of TIL expression in multiple spatially separate regions from a population of breast tumors. Though our patient cohort was relatively small, we demonstrated that, in this population, CD3, CD8, and CD20 show substantial heterogeneity but that heterogeneity is greatest within the core biopsy and to a lesser extent between biopsies of the same tumor. While this is a small study, our data suggests that a single core may be sufficient to estimate the TIL heterogeneity for an entire breast tumor. Future studies with larger patient populations with outcome data are needed to validate this observation.

## Abbreviations

CC, correlation coefficient; ER, estrogen receptor; DCIS, ductal carcinoma in situ; FOV, field of view; H&E, hematoxylin and eosin; HER2, human epidermal growth factor receptor 2; ICC, intraclass correlation coefficient; PD-1, programmed cell death receptor 1; PD-L1, programmed cell death receptor 1 ligand; QIF, quantitative immunofluorescence; TIL, tumor-infiltrating lymphocytes; TNBC, triple-negative breast cancer

## Additional file

Additional file 1: Figure S1. Batch to batch testing. Average AQUA® scores for serial sections of tonsil whole tissue run along with batch samples to ensure consistency in staining and analysis between batches. **Figure S2.** Distribution of CD20 in different FOVs from a single core on a single slide. CD20 cells often form tertiary lymphoid structures that give rise to highly heterogeneous FOVs on even a single slide. This is an illustration of a CD20 stain showing a tertiary lymphoid structure on the *left* and a nearby negative FOV on the *right*. **Figure S3.** Distribution of TIL subsets and intratumor heterogeneity in breast cancer. Representative immunofluorescence images showing the heterogeneity of CD3 (*red*), CD8 (*green*) and CD20 (*magenta*) in breast cancer tissue. Fluorescence signal was captured and unmixed using automated quantitative epifluorescence microscopy. Areas with high (*left panels*), intermediate (*center panels*), and low (*right panels*) signal for each marker obtained from the same core are shown although each row is from a separate patient for optimal illustration. **Figure S4.** Comparison of QIF versus cell count for CD8 and CD20. Graphs comparing cell counts with INform on the Y axis compared to QIF by AQUA® on the X-axis show a good, but not perfect correlation as the methods measure different tissue parameters. QIF AQUA® score is more similar to a protein concentration per unit area (FOV) while the cell count shows the absolute cell count in the same FOV (in this case a TMA spot from a lung cancer cohort). (PPTX 994 kb)
