# Supplementary material for: Quantitative assessment of the spatial heterogeneity of tumor-infiltrating lymphocytes in breast cancer
Source: Breast Cancer Res. 2016 Jul 29;18:78. doi: 10.1186/s13058-016-0737-x (PMC4966732; doi:10.1186/s13058-016-0737-x)
Supplement: Additional file 1: Figure S1. — Batch to batch testing. Average AQUA® scores for serial sections of tonsil whole tissue run along with batch samples to ensure consistency in staining and analysis between batches. Figure S2. Distribution of CD20 in different FOVs from a single core on a single slide. CD20 cells often form tertiary lymphoid structures that give rise to highly heterogeneous FOVs on even a single slide. This is an illustration of a CD20 stain showing a tertiary lymphoid structure on the left and a nearby negative FOV on the right. Figure S3. Distribution of TIL subsets and intratumor heterogeneity in breast cancer. Representative immunofluorescence images showing the heterogeneity of CD3 (red), CD8 (green) and CD20 (magenta) in breast cancer tissue. Fluorescence signal was captured and unmixed using automated quantitative epifluorescence microscopy. Areas with high (left panels), intermediate (center panels), and low (right panels) signal for each marker obtained from the same core are shown although each row is from a separate patient for optimal illustration. Figure S4. Comparison of QIF versus cell count for CD8 and CD20. Graphs comparing cell counts with INform on the Y axis compared to QIF by AQUA® on the X-axis show a good, but not perfect correlation as the methods measure different tissue parameters. QIF AQUA® score is more similar to a protein concentration per unit area (FOV) while the cell count shows the absolute cell count in the same FOV (in this case a TMA spot from a lung cancer cohort). (PPTX 994 kb) [file 13058_2016_737_MOESM1_ESM.pptx]

## Slide 1
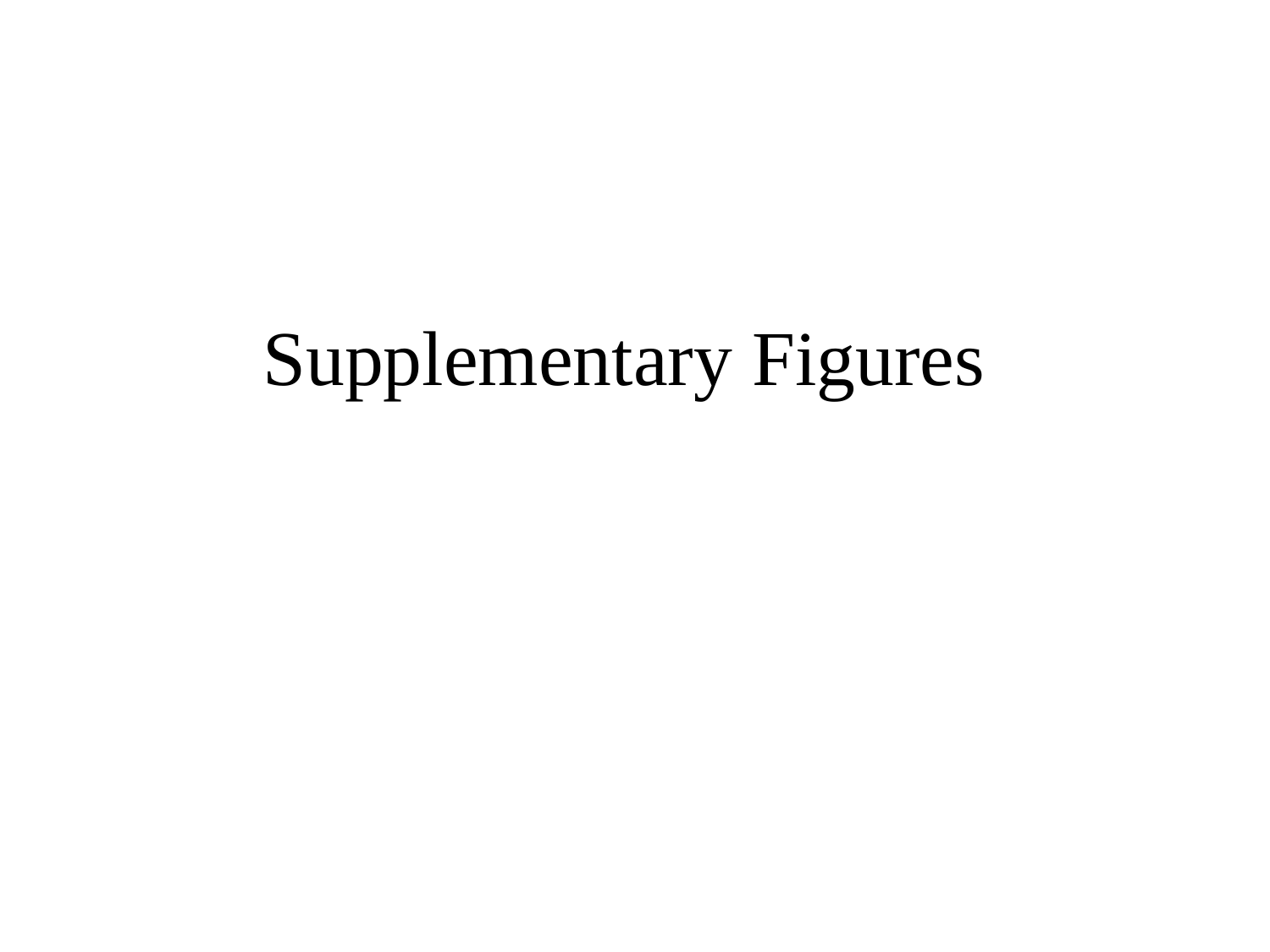

# Supplementary Figures

## Slide 2
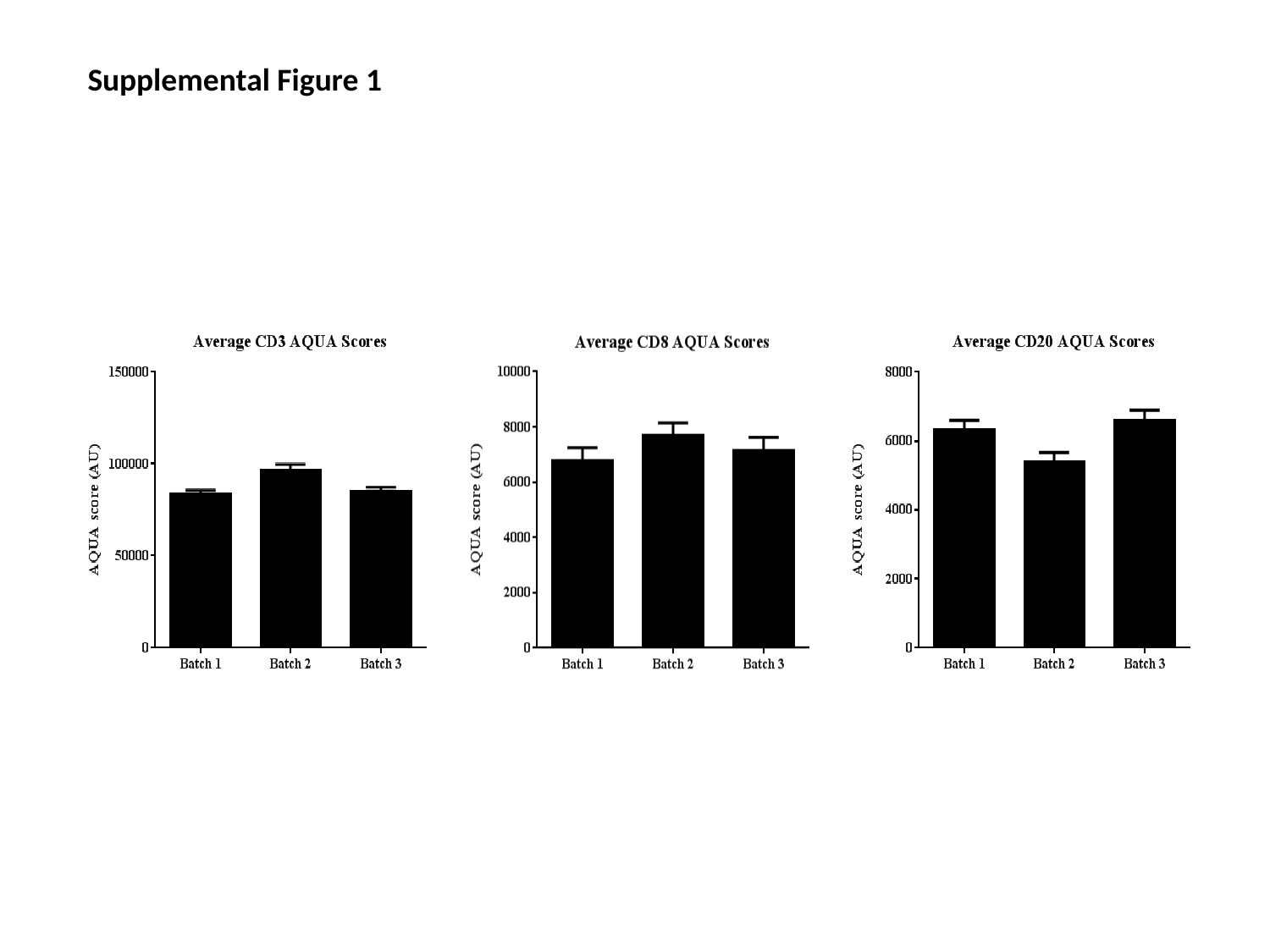

Supplemental Figure 1

## Slide 3
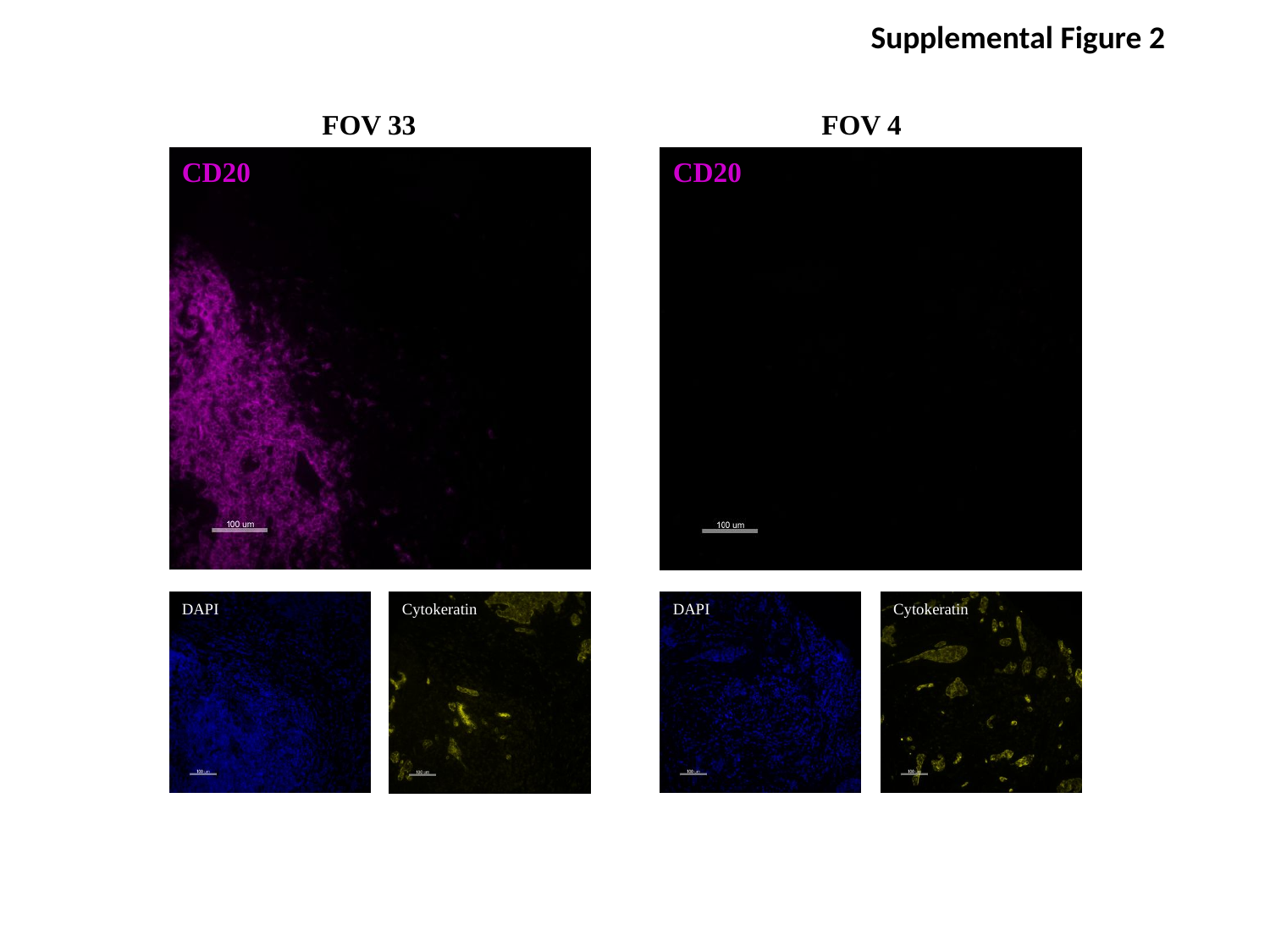

Supplemental Figure 2
FOV 33
FOV 4
CD20
CD20
DAPI
Cytokeratin
DAPI
Cytokeratin

## Slide 4
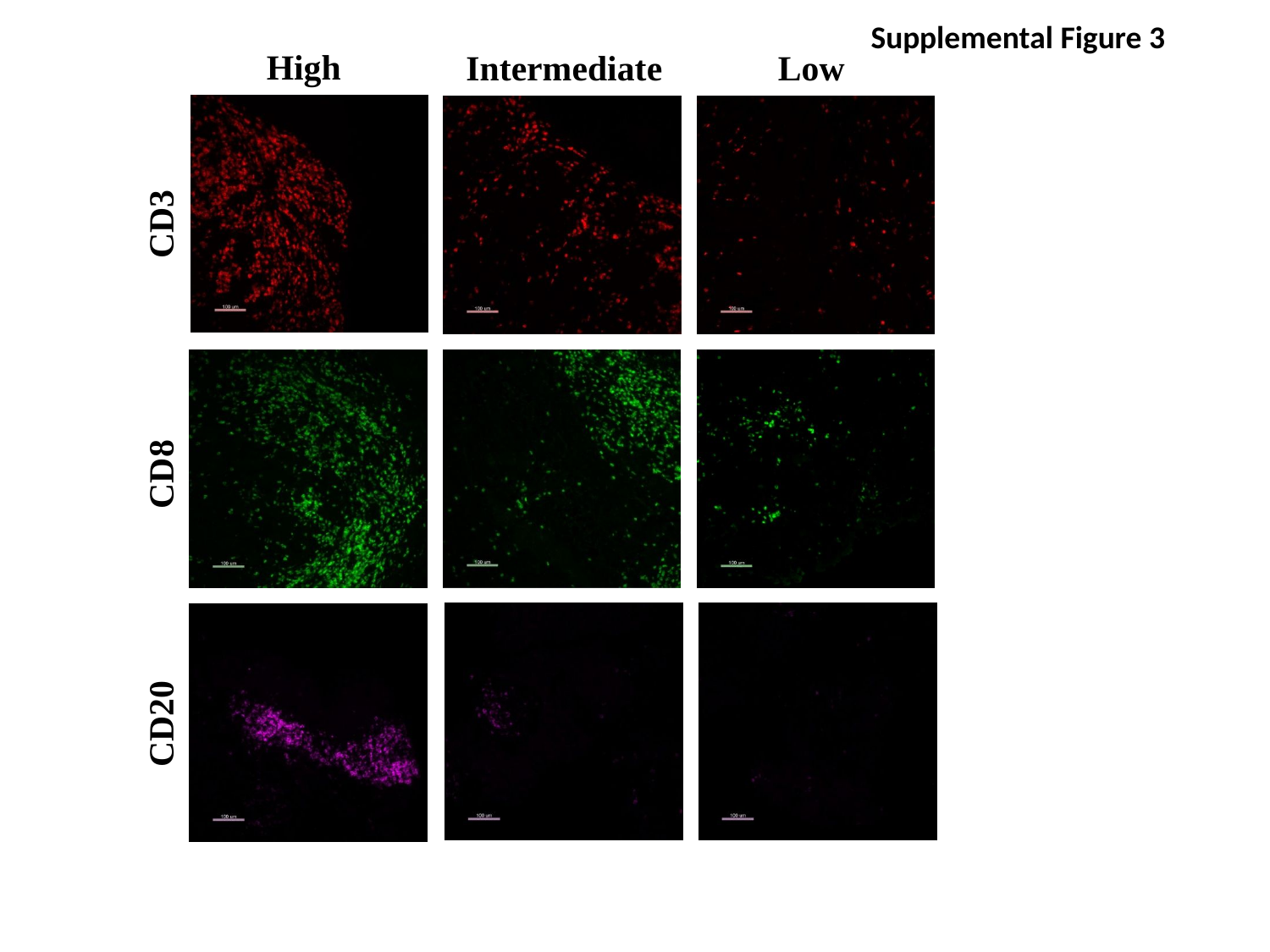

Supplemental Figure 3
High
Intermediate
Low
CD3
CD8
CD20

## Slide 5
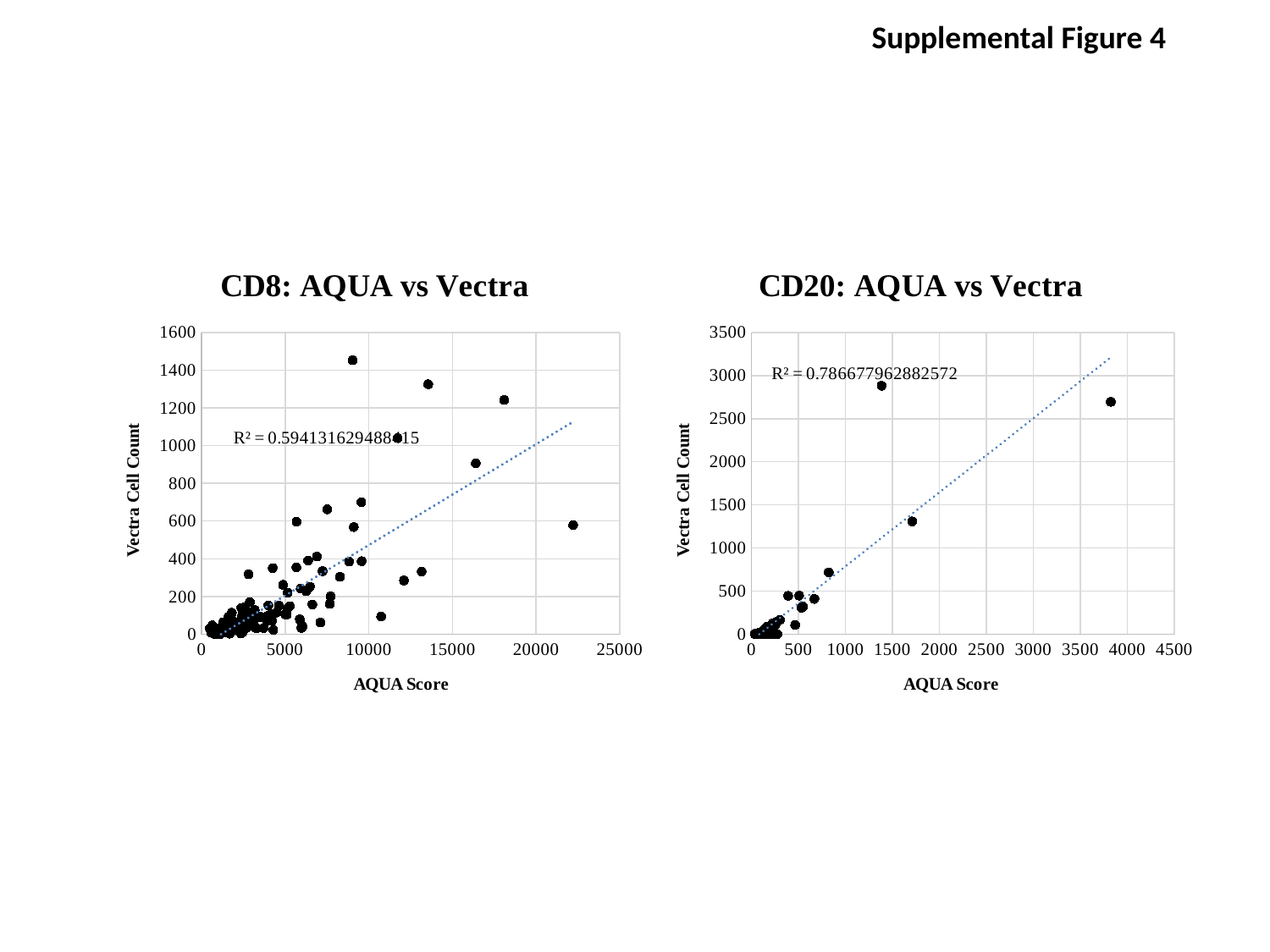

Supplemental Figure 4
### Chart: CD20: AQUA vs Vectra
| Category | |
|---|---|
### Chart: CD8: AQUA vs Vectra
| Category | |
|---|---|
